# Investigating individual differences in adult bilinguals’ spelling of cognates: An analysis of cross-linguistic effects

**DOI:** 10.1017/S1366728926101126

**Published:** 2026-04-15

**Authors:** Valeria M. Rigobon, Nuria Gutiérrez, Ashley A. Edwards, Laura M. Steacy, Donald L. Compton

**Affiliations:** 1 Australian Catholic University, Australian Centre for the Advancement of Literacy, Australia; 2 Florida Center for Reading Research, USA; 3 Department of Educational Psychology, University of Connecticut, USA; 4 Florida State University Department of Psychology, USA

**Keywords:** bilingualism, spelling, cognates, cognate facilitation effect, individual differences

## Abstract

Examining 62 college students who are bilingual in Spanish and English, this study assessed key predictors of irregular English word spelling accuracy, including cognates and non-cognates. Explanatory item response models tested the contributions of word-level (e.g., orthographic similarity [OS] and phonemic similarity [PS] between English and Spanish word forms) and person-level predictors (e.g., literacy skills in English and Spanish) to item-level spelling accuracy. In line with prior investigations of cognate spelling in English, spelling accuracy was predicted by generally stronger English decoding skill and higher OS, with no significant influence of Spanish abilities. However, OS effects diminished after removing identical cognates from the outcome variable. An exploratory analysis revealed similar effects of English and Spanish decoding fluency on the likelihood of non-cognate spelling accuracy. These results have implications for understanding how orthographic representations of cognates are stored and accessed in the bilingual lexicon, particularly in alphabetic orthographies.

## Highlights

- Bilingual adults spelled 60 irregular words in English (cognates and non-cognates).
- Competing with other English literacy skills, Spanish skills did not impact spelling.
- Orthographic similarity (OS) facilitated spelling accuracy.
- OS effects diminished when identical cognates were removed from analyses.
- Spanish and English decoding skills similarly influence non-cognate spelling accuracy.

To understand how knowledge of two languages can interact in the bilingual mental lexicon, **cognates** (i.e., words that share the same meaning, similar spellings and sometimes similar pronunciations between languages) have been used to test hypotheses about the organization and activation of information from different languages when listening, reading and speaking in each language of proficiency. Cognate representations have been widely investigated with bilingual children and adults across behavioral studies of speech production (Schwartz et al., 2007), translation (de Groot, 1992; Muscalu & Smiley, 2019; Sánchez-Casas et al., 1992), lexical decision (Arana et al., 2022; Carrasco-Ortiz et al., 2021; Comesaña et al., 2012; Comesaña et al., 2015; Dijkstra et al., 1999; Lemhöfer & Dijkstra, 2004) and spelling (Iniesta et al., 2021; Rigobon et al., 2023). Mixed findings of facilitation and inhibition from activating L1 representations in L2 cognate tasks suggest that the benefits of L1 knowledge are not universal. Instead, they appear contingent on specific cognitive demands, varying degrees of orthographic and phonological overlap, and particular levels of bilingual proficiency.

However, the mechanisms by which activation of sublexical or lexical representations from one language may facilitate the formation and recall of orthographic representations in another language are still not clearly understood in the context of **spelling,** one of the most challenging tasks to master in literacy development (Bosman et al., 2006). The current study aims to investigate how different cognate conditions affect the probability of English spelling accuracy among bilingual adults. For two key reasons, we draw from a convenience sample of heritage Spanish speakers (i.e., bilinguals who informally acquired Spanish oral language skills through home exposure) who are also skilled in English speaking, reading and writing. First, from a practical standpoint, Spanish speakers are the largest heritage language community in the United States (Kutlu & Kircher, 2021) and the largest growing bilingual community in the state where the study was conducted. Second, given that heritage speakers vary in the degree that they retain or expand representations of words in Spanish as they become more dominant in and reliant on English throughout university studies (including advanced reading and writing for academic purposes), this sample presents an opportunity to examine how individual differences in Spanish proficiency may influence the degree to which cognate effects are facilitatory versus inhibitory on a linguistically demanding task. Specifically, we examine word-level variables like the amount of orthographic similarity (OS) and phonemic similarity (PS) between Spanish and English cognate forms and person-level variables such as Spanish and English proficiency in word reading, decoding and vocabulary. The task includes spelling of English-Spanish identical cognates (e.g., *arsenal*), non-identical cognates (e.g., *pivot-pivote*) and non-cognates (e.g., *appraisal-tasación*) in English.

## 1. Conditions for cognate effects in written production

Previous studies of bilingual adults’ written translation (Muscalu & Smiley, 2019) and spelling (Iniesta et al., 2021; Rigobon et al., 2023) have provided evidence of lexical influences (e.g., knowledge of words in one language competing with whole-word representations in another language during word recognition) and sublexical influences (i.e., knowledge of spelling patterns from one language influencing recall of word spelling in another language) from one language to another. Muscalu and Smiley’s (2019) results from typed word translations of Russian-English bilingual adults showed shorter response onset latencies for cognates compared to non-cognates, suggesting that lexical retrieval was facilitated by a word’s overlap in spelling between Russian and English (i.e., cognateness). At the sublexical level, however, cognate *inhibition* effects were evident in longer latencies to complete word translations and higher error rates in spelling accuracy for cognates compared to non-cognates. These results support a model of cross-language processing in which a bilingual’s retrieval of a word’s translation equivalent is facilitated for cognates, followed by interference in the production of that word’s spelling, suggesting that facilitation at one level of processing does not necessarily continue into later stages of processing. These findings align with assumptions from MacKay’s (1987) interactive model: cross-language activation occurs “in both top-down and bottom-up directions, with selection of a lexical item influenced by activation” from the semantic and phonological systems, as well as the orthographic system (Muscalu & Smiley, 2019, p. 850).

Using a traditional word dictation task, Iniesta et al. (2021) reported evidence of a cognate interference effect in English spelling with cognates being spelled less accurately and typed more slowly (based on response onset time) than non-cognates in English-Spanish bilingual adults. Interestingly, cognates did not significantly differ from non-cognates in latency for typing the rest of the word; together, these findings differ from the cognate facilitation effects reported by Muscalu and Smiley (2019) with their sample of Romanian-English bilinguals and a different task. Additionally, Iniesta et al. found that different categorical measures of cognateness (e.g., whether a word is classified as having high versus low OS based on a cutoff value) influenced the likelihood of facilitation in response latency and accuracy. Cognates with higher phonological^1^ similarity (PS) were retrieved more quickly (i.e., faster key stroke for the first letter) and spelled more accurately compared to cognates with lower PS. The PS effect on spelling accuracy for the rest of the word (after the first key) was found for words with lower OS, but not for words with higher OS, suggesting that PS influences initial lexical access regardless of OS, but only impacts sublexical processing of cognates with lower OS.

Building on the debate over whether cognateness consistently facilitates spelling across different levels of language proficiency, a recent study by Rigobon et al. (2023) provided evidence for cognate facilitation in spelling irregular English words (i.e., words containing one or more graphemes whose spellings cannot be derived directly from the most frequent phoneme-to-grapheme mappings in English) that are more orthographically transparent in Spanish (e.g., *inseparable*). The study demonstrated that English–Spanish bilinguals showed greater spelling accuracy on cognates (compared to non-cognates) as the OS and PS between Spanish and English increased, regardless of whether cognateness was measured categorically (e.g., words categorized as cognate versus non-cognate) or continuously (e.g., words’ degree of OS and PS between English and Spanish forms). In contrast, English-only monolinguals’ ability to spell cognates was not affected by cognateness. Because the stimuli in that study were primarily orthographically identical or near-identical cognates and PS was not systematically controlled for during stimuli selection, it remains unclear how inclusion of non-identical cognates with lower OS or PS in the spelling task stimuli might facilitate or inhibit bilinguals’ English spelling accuracy.

Insights from studies of bilinguals’ performance on lexical decision tasks further highlight the complexity of cognate effects, suggesting that substantial orthographic overlap drives facilitation of response speed. Arana et al. (2022) and Comesaña et al. (2015) reported that significant facilitatory cognate effects were restricted to identical cognates and the stimuli list composition determined whether response speed was facilitated or inhibited by a word’s cognateness. Arana et al. (2022) found that cognates were more quickly recognized compared to non-cognates, but only when a substantial proportion of the stimuli list consisted of orthographically identical cognates. With those items reduced to less than half of the composition or fully removed, recognition speed of cognates was slower compared to non-cognates, suggesting that OS effects on cognate processing are not equal for identical and non-identical cognates. This effect of stimuli list composition has not yet been tested in spelling accuracy.

## 2. Lexical quality in the bilingual lexicon

Cognates are one of many shared linguistic features that a bilingual individual may recognize between one language (e.g., Spanish) and another (e.g., English; Connor, 1996; Ramirez et al., 2013; Verhoeven, 2017). Accumulation of these cross-linguistic observations can support the application of linguistic knowledge from Spanish to English to create a “boosting” effect for bilingual students’ sensitivity to individual English sounds and how they map onto English print when constructing orthographic representations (see Kremin et al., 2019). This boosting effect in sublexical sensitivity and knowledge directly aligns with two key assumptions of the lexical quality hypothesis (LQH, Perfetti & Hart, 2001, 2002), which states that a fully specified orthographic representation in the reader’s lexicon should build over time and experiences in both precision (i.e., the specific letters and their position within the word) and redundancy (i.e., the various pronunciations possible for that word), allowing for more fluent and accurate reading and spelling.

The first key assumption of the LQH is that only one set of grapheme-phoneme correspondences (GPCs) is required for *building* the orthographic representation of a word, but two sets of GPCs may be a “safety net” (i.e., greater redundancy) that boosts the probability of fluent word reading in various contexts, and possibly, more accurate spelling over time. If having two or more pronunciations stored or accessible for a given English word contributes to the formation of a more precise orthographic representation of the word, then perhaps bilinguals who have some proficiency in a more transparent orthography like Spanish can draw upon phonological representations activated in Spanish, either consciously or subconsciously, to supplement an incomplete representation of the English form during spelling. In the case of a cognate in Spanish and English, the individual may benefit from analyzing an existing phonological representation in Spanish with more transparent sublexical units that can map directly onto the more ambiguous sublexical units in the English spelling. Consider the example of the irregular English word *ritual*, a cognate with identical spelling and meaning in Spanish and English. Each phoneme in Spanish can only correspond to one possible grapheme (except for the /r/ sound, usually represented as *rr* but is spelled with a single *r* at a word’s onset), whereas several phonemes in English can each correspond to multiple graphemes (e.g., /tʃ/ to the graphemes *t, tch* or *ch* [as in *mature, pitch*, and *rich*]; the schwa in the second syllable to any vowel letter), making the units in the Spanish translation easier to spell correctly based on basic phoneme-grapheme correspondences (PGCs). This example illustrates the possibility of the redundancy principle extending to knowledge of how a cognate is pronounced in more than one language, with both languages being alphabetic orthographies, contributing to the formation of a high-quality orthographic representation. A test of this assumption would require bilinguals to spell cognates in English that (a) contain more opaque PGCs in English and more transparent PGCs in Spanish and (b) are familiar in Spanish oral vocabulary.

The second key assumption is that in the absence of fully specified representations, individuals are often forced to rely on other sources of information (e.g., most frequent PGCs and general spelling rules and patterns) to aid in word spelling. However, resorting to the most frequent PGCs or spelling generalizations can still lead to error-filled spelling given the opaque and inconsistent nature of English (particularly for vowels in irregular words). While the lexical quality hypothesis describes a monolingual speller’s sources of information, for a bilingual speller, it may be possible to recruit general knowledge of Spanish PGCs to fill in the gaps in an impoverished orthographic representation in English. One way to test this is by asking bilinguals to spell irregular English words that cannot be spelled correctly based on application of most frequent PGCs in English, which forces participants to rely on a strategy to supplement a poor orthographic representation of the word if they cannot confidently recall its spelling from memory.

To expand the field’s understanding of word-level characteristics, person-level features and task demands that impact cognate facilitation in recall of orthographic representations, the current study explored effects of OS, PS and general literacy skills in English and Spanish on bilinguals’ spelling accuracy of irregular English words, including cognates and non-cognates. Instead of relying on subjective self-reports like age of acquisition, language dominance and language proficiency – which have been shown to impact the strength and likelihood of cognate facilitation – the current study used objective test scores of vocabulary, decoding and word reading in both languages to predict spelling accuracy.

## 3. The current study

**Figure 1.**
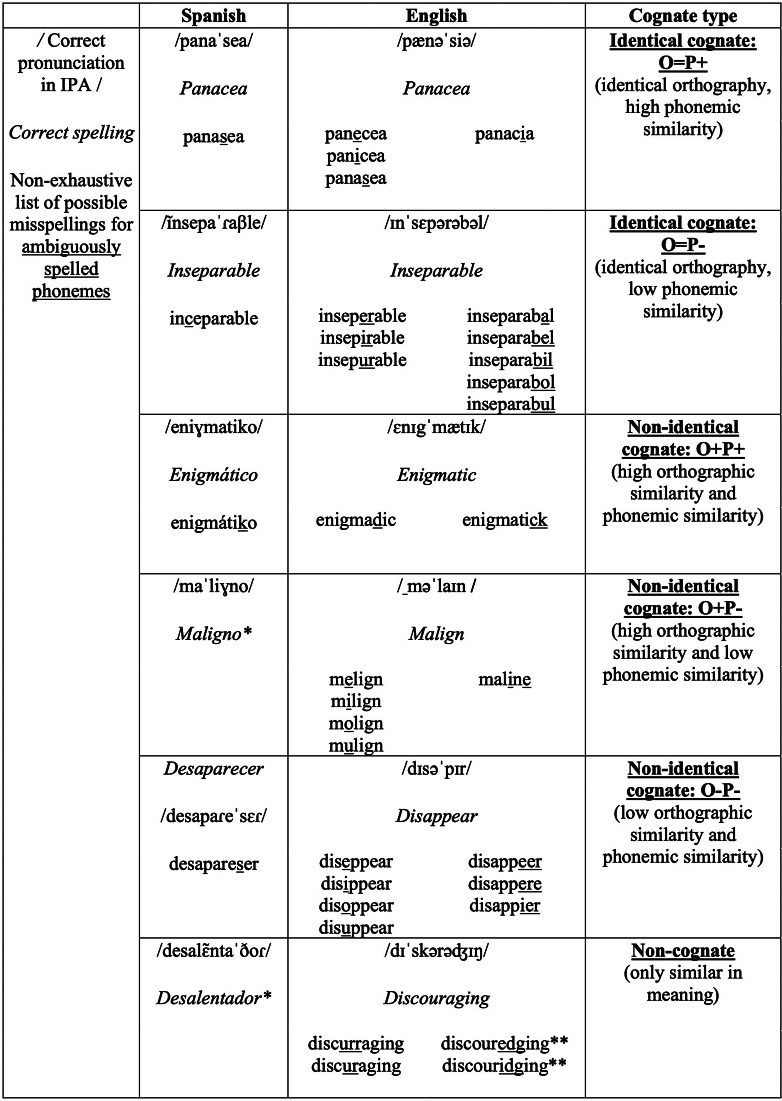
Comparison of cognate and non-cognate words in Spanish and English from the current study’s stimuli. *Note:* *These Spanish words contain 0 ambiguously spelled phonemes, and therefore, no alternate word spellings are listed below the correct spelling. **These misspellings of *discouraging* include alternate spellings for both the *a* vowel grapheme and the *g* consonant grapheme.

Bilingual adults were assessed on their spelling of cognates (varying in OS and PS between Spanish and English) to identify individual differences related to word characteristics and participants’ proficiency in literacy skills in English and Spanish. Cognates were characterized as translation equivalents (i.e., similar or identical meaning) between Spanish and English with five different possible categorizations based on OS and PS between the Spanish and English forms of the words (see Figure 1).

This study was carefully designed to answer questions about varying levels of OS and PS and individual differences in language proficiency that have not been addressed by previous work with bilingual adults’ spelling:

1. Does the interaction of continuous OS and PS measures impact the likelihood of bilinguals’ English word spelling accuracy?
2. Are the facilitatory effects of OS on bilinguals’ English word spelling accuracy restricted to identical cognates?
3. Which specific literacy-related skills (i.e., decoding fluency, word reading fluency and expressive vocabulary) in Spanish and English predict English word spelling accuracy?

At the word-level, the first hypothesis is that bilingual adults’ likelihood of cognate spelling accuracy will vary significantly as a function of the interaction between continuous measures of OS and PS while accounting for two word features known to influence spelling difficulty among adults (e.g., Bonin et al., 2013; Delattre et al., 2006; Rigobon et al., 2023, 2024): frequency and degree of irregularity. Specifically, spelling accuracy of cognates with lower OS will be more sensitive to effects of PS, aligning with Iniesta et al.’s (2021) findings of categorical OS and PS effects on spelling accuracy. The second hypothesis, based on prior findings from lexical decision studies (Arana et al., 2022; Comesaña et al., 2015), is that the facilitatory effect of OS will diminish when identical cognates are removed from the analysis.

At the person level, the third hypothesis is that bilinguals’ ability to capitalize on Spanish knowledge for facilitated spelling of English cognate forms will vary significantly as a function of Spanish proficiency as follows: higher performance on Spanish nonword reading fluency (i.e., measure of ability to automatically apply transparent GPCs) will predict higher likelihood of item-level English spelling accuracy, while accounting for general English literacy skills typically associated with adults’ spelling performance (e.g., Ocal & Ehri, 2017a; Rigobon et al., 2023, 2024): word reading fluency, pseudoword reading fluency, expressive vocabulary and overall familiarity with the target spelling words. Based on the LQH (Perfetti & Hart, 2002), support for this hypothesis would suggest that adults who have stronger representations of sublexical units in Spanish are more likely to show facilitation from activated Spanish word representations in the context of spelling irregular English words compared to peers with weaker sublexical representations in Spanish. We include measures of Spanish real word reading fluency, spelling, expressive vocabulary and overall familiarity with the target spelling words’ translation equivalents in Spanish as exploratory cross-linguistic predictors of English spelling accuracy.

## 4. Methods

### 4.1. Participants

Over the course of 14 months, data were collected from 82 undergraduate students in the psychology subject pool of a large public university in the Southeast region of the United States. Table 1 provides demographic information and descriptive statistics of the participants. Prior to the study’s initiation, ethical approval was obtained from the review committee for human subject research, in compliance with the U.S. Federal Policy for the Protection of Human Subjects. Subjects signed up online for two individual remote testing sessions of 45–60 minutes each on Zoom, and consent to participate with video and/or audio recording of the sessions was obtained at the beginning of the first session. Participants were compensated with extra credit for selected courses.

**Table 1.** Demographic characteristics and descriptive statistics of the sample (*N* = 62)

Age (Years)	*M* = 20.42 *SD* = 3.66
Female	74%
Hispanic/Latine	98%
White	84%

	Maximum raw score	*M*	*SD*
Outcome variable			
Dependent spelling	60	41.68	8.15
Person-level variables			
En decoding acc	32	28.48	2.71
Sp decoding acc	34	29.19	2.73
En familiarity	60	52.18	6.57
Sp familiarity	60	12.92	5.54
En word reading acc	76	70.39	3.46
Sp word reading acc	76	69.55	6.91
En vocabulary	44	29.90	5.15
Sp vocabulary	44	26.42	7.59
En spelling	59	50.44	3.56
Sp spelling	59	36.13	9.73
En decoding fluency	66	53.35	7.71
Sp decoding fluency	42	30.88	9.86
En word reading fluency	108	86.52	13.71
Sp word reading fluency	42	38.73	12.15

Self-reported spelling strategy usage^a^	Visual. word (En)	Visual. word (Sp)	Sounding out word (En)	Sounding out word (Sp)	Word analogy (En)	Morph. Analysis (En)
	82%	44%	77%	39%	1%	39%

*Note:* Raw scores are reported for all measures. *M* and *SD* are used to represent mean and standard deviation, respectively. En and Sp are used to represent whether the measure was of English or Spanish performance, respectively. Acc = Accuracy. Fluency represents number of words read correctly (i.e., word reading) or pseudowords read correctly (i.e., decoding) in 45 seconds. Visual = Visualizing; Morph. = Morphological. Percentages are rounded to the nearest whole number.

a Strategy usage percentages represent the proportion of the total sample that reported using this strategy, with the option to choose more than one strategy.

After signing up for their first session, subjects were sent a link to the online Language History Questionnaire 3.0 (LHQ3; Li et al., 2020) to complete asynchronously on their own device within 2 days of signing up for participation and determine their eligibility for later testing. Subjects answered a range of questions about their daily and historical usage of Spanish and English (e.g., age at which they started listening, speaking, reading and writing in each language) and self-perceived proficiency on reading, speaking, spelling and writing in each language. Based on responses, those who met the inclusion criteria of (a) being 18 years or older, (b) having acquired Spanish listening and speaking skills before or during acquisition of English listening, speaking, reading and writing skills (i.e., heritage Spanish speakers) with no third language exposure or proficiency, and (c) having very good or excellent abilities in English listening, speaking, reading and writing skills were granted their first research credit and contacted to confirm the date and time for their first testing session. Those who completed the questionnaire but did *not* meet the inclusion criteria (e.g., reported learning a third language or learning Spanish several years after beginning to learn English) were awarded research credit for their participation in the screening survey, but were not scheduled for further testing. In response to preliminary participant feedback indicating LHQ3 completion times exceeding the initially advertised 30-minute duration, the protocol was modified to include an alternative participation option. Participants were offered the choice between completing the full questionnaire for maximum study credit or answering three screening questions to verify inclusion criteria at the beginning of their first testing session for partial credit. This modification was implemented to minimize participant burden and retain participants while maintaining data quality and ethical research practices.

There was an intentional oversampling for Hispanic/Latine student populations to recruit more heritage Spanish speakers who would meet the eligibility requirements. The participants’ ages ranged from 18 to 41 years. The heterogeneity of the sample is reflected in the wide range of performance on measures of general English and Spanish vocabulary, word reading and decoding fluency, untimed word reading accuracy and spelling.

### 4.2. Procedures

During the first online testing session, participants were assessed on all Spanish measures. In the second online testing session (scheduled at least 2 days after first session), the target word familiarity and dependent English spelling tasks were administered first, followed by all remaining English measures and a final demographics survey. Raw total scores were calculated for each measure assessed in both testing sessions. Item-specific accuracy scores (0 = incorrect, 1 = correct) were recorded on pen and paper for the dependent spelling measure.

### 4.3. Dependent spelling measure and embedded item-specific familiarity

*English spelling.* The experimental spelling task, based on procedures used in the Woodcock-Johnson III (Schrank, 2005) standardized spelling task, was designed to measure participants’ ability to spell 60 irregular cognate and non-cognate words in English correctly administered in randomized order (see Appendix A). Cognates in Spanish and English were selected from the CogNet database (Batsuren et al., 2019, 2021). Next, print frequencies of the English forms from the filtered CogNet list were searched in the English Lexicon Project database (ELP; Balota et al., 2007). For nouns and adjectives that had English print frequency values available, print frequencies of their Spanish forms were searched in the EsPal database (Duchon et al., 2013). Of the remaining cognates with Spanish frequency data available, OS and PS were calculated for each pair of translation equivalents using objective computations of normalized orthographic Levenshtein distance (OLD) and phonological Levenshtein distance (PLD) values. Although OS and PS were modeled as continuous predictors of spelling accuracy for the main analyses described below, words were categorized into groups in this initial procedure to ensure that the selected words represented a wide range of possible combinations of orthographic and phonological features. High and low phonemic and orthographic similarity were defined by median split to create O-/O+ and P-/P+ distinctions that allowed for at least 10 different cognates to fall into each of the five cognate categories exemplified in Figure 1: O=P+ (identical orthography, high PS), O=P- (identical orthography, low PS), O+P+ (high OS and PS), O+P- (high OS, low PS), O-P- (low OS and PS). Parameters of the final drafted cognate list (*n* = 50) were entered into the ELP to restrict the search for 10 non-cognates with matching average length, print frequency, number of orthographic/ phonological neighbors and part of speech. Selected non-cognates were filtered through the CogNet database to check that none of them were cognates or false cognates. See Table 2 for descriptive statistics of the task items’ word-level characteristics.

**Table 2.** Descriptive statistics of word-level characteristics for the dependent spelling words

Word-level variables	Identical Cognates (*n =* 20)		Non-Identical Cognates (*n =* 30)		Non-Cognates (*n =* 10)		*F*(1, 60)	*p*
	*M*	*SD*	*M*	*SD*	*M*	*SD*		
En frequency	7.20	1.69	6.70	1.69	6.39	.56	1.04	.36
Sp frequency	.84	.56	.72	.55	.96	.48	.79	.46
En length	7.85	1.76	8.90	2.02	9.00	1.15	2.33	.11
Sp length	7.85	1.76	8.60	1.89	8.70	2.63	1.03	.36
En morphemes	1.80	.95	1.83	.79	1.90	.74	.05	.95
En phonemes	7.00	1.65	7.53	1.93	6.70	1.25	1.09	.34
En syllables	2.85	.59	3.23	.97	2.60	.52	2.89	.06
N of Schwas	1.20	.41	1.20	.71	1.20	.92	0.00	1.00
En OLD20	2.93	.65	3.39	.93	3.39	.50	2.23	.12
En PLD20	3.11	1.03	3.38	1.14	3.05	.63	.60	.55
En ONS	.30	.66	.27	.64	.20	.42	.09	.92
Sp ONS	.65	1.09	1.17	1.90	3.10	7.40	1.90	.216
En PNS	1.25	2.97	.70	1.78	.50	.85	.68	.51
Sp PNS	7.70	1.87	8.50	1.80	8.40	2.80	1.00	.37
OS	1.00	0.00	.65	.17	.16	.10	145.40	<.001
PS	.45	.14	.35	.14	.10	.16	14.74	<.001

*Note: M* and *SD* are used to represent mean and standard deviation, respectively. ANOVA Bonferroni correction was conducted to correct for multiple comparisons at the word level (3 groups). En = English; Sp = Spanish; OLD20 = Orthographic Levenshtein Distance 20 (the average number of transformations required through grapheme deletions, insertions or substitutions to change a word into its 20 closest orthographic neighbors); PLD20 = Phonological Levenshtein Distance 20 (same as OLD20, but with phoneme transformations required to change a word into its 20 closest phonological neighbors); ONS = Orthographic Neighborhood Size; PNS = Phonological Neighborhood Size; OS = Orthographic Similarity; PS = Phonemic Similarity.

At the beginning of the second testing session, testers guided the participants to share their screen and disable their spell check before beginning the spelling task online. Participants were instructed to play a recording of an example item and repeat it back to the tester to ensure that they heard the word correctly.^2^ Participants then answered a multiple-choice question about whether the word sounded familiar (0 = no/unsure, 1 = yes), played the recording of the word again and typed their spelling of the word, scored dichotomously based on accuracy (0 = incorrect, 1 = correct). After typing their response, participants answered another multiple-choice question about whether they knew how to spell the word from memory, used a strategy to help them spell the word or guessed the word’s spelling. After the example item, participants followed the same procedure for the remaining items. Ordinal alpha for spelling was .89.

### 4.4. Independent word-level measures

*Cognateness.* Target spelling words were categorized as a non-identical cognate, identical cognate or non-cognate. Non-identical cognates do not share exact spelling overlap between Spanish and English translation equivalents. Word features between identical, non-identical and non-cognates were checked for group differences to confirm that they only differed significantly in OS and PS (see Table 2).

*Frequency.* Target spelling words’ log-transformed HAL frequency values, based on the Hyperspace Analogue to Language corpus, were taken from the ELP (Balota et al., 2007). The log-transformed HAL frequency reported for the list of target words ranges from 3.64 to 11.31.

*Number of schwas.* This measured the number of unstressed vowels (i.e., schwas) in each English word in the dependent spelling measure as a form of word irregularity that was not captured by the lexical features controlled for in the stimuli selection procedure.

*Orthographic similarity (OS).* OS was calculated for each word in the dependent spelling measure and its Spanish translation equivalent with objective computations of normalized orthographic Levenshtein distance. These scores accounted for maximum word length of the two words being compared and the minimum number of insertions, deletions and substitutions needed for the two words to be spelled identically. Identical cognate pairs in the O = conditions were scored as 1.

*Phonemic similarity (PS).* PS scores were calculated for the DISC phonetic transcriptions of each word in the dependent spelling measure and its Spanish translation equivalent^3^ with objective computations of normalized phonetic Levenshtein distance. English transcriptions were sourced from the Celex database (Baayen et al., 1993), and Spanish transcriptions were sourced from the B-PAL database (Davis & Perea, 2005).

### 4.5. Independent person-level measures: English and Spanish

*Familiarity.* This measure was adapted from a child-appropriate measure of polymorphemic words (Kearns et al., 2016) to test participants’ familiarity with the 60 words from the dependent spelling measure. English familiarity was embedded in the second testing session’s dependent spelling task, whereas Spanish familiarity was assessed separately at the end of the first testing session with translation equivalents of the 60 spelling words and 15 foil words. Participants were given similar instructions to listen to recordings of the Spanish words and indicate whether or not they had ever heard those words before the testing session. Responses in each measure were scored (0 = unfamiliar/not sure, 1 = familiar); each total score was calculated from the sum of items that were familiar. Ordinal alpha was .94 for English and .92 for Spanish.

*Vocabulary.* The picture vocabulary subtests from the Woodcock-Johnson III (Schrank, 2005) and Woodcock-Muñoz III (Muñoz-Sandoval et al., 2009) required participants to recognize and label up to 44 pictures with single word descriptions in English and Spanish, respectively. Responses in each measure were scored 0 for incorrect and 1 for correct; each total score was calculated from the sum of correctly identified items. The WJ-III and WM-III authors report a reliability coefficient of .93 for Spanish (based on calibration data, see Schrank, 2005) and split-half reliability of .81 for English.

*Decoding fluency.* In the phonemic decoding efficiency subtest from the TOWRE-2 (Torgesen et al., 2012), participants were asked to read a list of 66 nonwords as quickly and accurately as possible within the span of 45 s. A Spanish pseudoword reading task was adapted from the Test of Reading and Writing in Spanish (LEE; Defior Citoler et al., 2006), which required participants to read 32 nonwords as quickly and accurately as possible; the time taken to read the entire list was recorded. Responses in each measure were scored 0 for incorrect and 1 for correct, and each total score was calculated from the sum of correctly read items. Fluency scores were calculated by dividing the accuracy score by 45 to represent the number of correctly read nonwords per 45 seconds. The TOWRE-2 and LEE authors reported an alternate forms reliability of .92 for English and a test–retest reliability of .88 for Spanish.

*Spelling.* The spelling subtests from the Woodcock-Johnson III (Schrank, 2005) and Woodcock-Muñoz III (Muñoz-Sandoval et al., 2009) were used to measure general spelling ability of 59 increasingly difficult English and Spanish words, respectively. Responses in each measure were scored 0 for incorrect and 1 for correct according to American English spelling and the Royal Spanish Academy’s rules for Spanish spelling, respectively. Total scores were calculated from the sum of correctly spelled items. Before beginning the task, testers guided the participants to share their screen and disable their spell check. Participants were instructed to play a recording of an example word (including the word in a sentence and a repetition of the single word) before typing their spelling of the word. For the Spanish task, participants received the additional instruction of how to represent accented letters (i.e., letter followed by a ` symbol) to avoid the need for access to or familiarity with a Spanish keyboard. The WJ-III and WM-III authors reported a reliability coefficient of .93 for Spanish and split-half reliability of .90 for English.

*Word reading fluency.* The English sight word efficiency subtest from the TOWRE-2 (Torgesen et al., 2012) required participants to read a list of 108 sight words as quickly and accurately as possible within the span of 45 s for each list. An adapted measure of the Spanish sight word reading subtest from the Test of Reading and Writing in Spanish (LEE; Defior Citoler et al., 2006) required participants to read a list of 42 words as quickly and accurately as possible; the time taken to read the entire list was recorded. Responses in each measure were scored 0 for incorrect and 1 for correct; each total score was calculated from the sum of correctly read items. Fluency scores were calculated by dividing the total accuracy score by 45 to represent number of correctly read words per 45 seconds. The TOWRE-2 and LEE authors reported an alternate forms reliability of .92 for English and a test–retest reliability of .88 for Spanish.

### 4.6. Fidelity

A fidelity-of-implementation checklist was developed based on the testing scripts for the Test of Word Reading Efficiency (TOWRE-2; Torgesen et al., 2012), Test of Reading and Writing in Spanish (Defior Citoler et al., 2006), Woodcock-Johnson III (Schrank, 2005), Woodcock-Muñoz III (Muñoz-Sandoval et al., 2009) and researcher-created scripts for the remaining measures. Undergraduate research assistants were trained to administer testing sessions with high fidelity via weekly Zoom training sessions over the course of 2–3 months, after which each assistant was required to practice administering each measure to the primary investigator or another trainer. Finally, research assistants completed mock remote test administration sessions with the trainer, who addressed all discrepancies after the completed session. In the rare event that fidelity of implementation was <80% during the mock session, the research assistant received targeted feedback, practiced more and completed another mock remote testing session with >80% fidelity. The trainers met with each assistant after their first testing session to address any unplanned complications with subjects and answer any questions about testing. Testing sessions were video and audio recorded on Zoom. All tests (excluding auto-summed familiarity scores on Qualtrics) were double-scored by three team members, and all scores were double-entered for higher reliability; a separate researcher was asked to resolve any discrepancies between each set of scores and entries. The REDCap (Research Electronic Data Capture) tool hosted at Vanderbilt University (Harris et al., 2009) was used to enter and manage data throughout the study period.

### 4.7. Data analytic procedures

Item-response based crossed random effects models were used to simultaneously account for the role of different predictors of item-level target word spelling variance. These cross-classified models were used to predict participants’ spelling accuracy of the specific word (e.g., *accelerator*), coded as a dichotomous response (1 = correct, 0 = incorrect), using word-level (e.g., English written word frequency of *accelerator*), person-level (e.g., English vocabulary total score) and interaction (e.g., OS by PS) predictors. Person-by-word level interactions (e.g., Spanish decoding fluency score by cognateness) were also tested in exploratory models. All analyses were conducted using a binomial distribution with a logit link, available through the glmer function in the lme4 package (Bates et al., 2015) from R programming (R Development Team, 2012). All continuous person and word predictors were grand mean-centered to aid in interpreting the intercept and coefficients.^4^ Variance explained for person and word for each model was calculated by determining the reduction in person and word variance from the unconditional model. The formula was 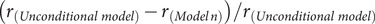, where *n* represents the model to which the unconditional model was compared (Bryk & Raudenbush, 1992).

## 5. Results

### 5.1. Missing data

Out of the 82 participants who completed the first testing session, 66 completed the second session. Despite all participants self-reporting that they had good abilities in English listening, speaking, reading and writing, four outliers were identified based on their English vocabulary scores, which were more than two standard deviations below the rest of the sample (*M =* 28.17, *SD =* 8.52), and were removed from subsequent analyses. All analyses were completed with data from the 62 remaining participants.

### 5.2. Descriptive statistics

Word-level descriptive statistics for the 50 cognates and 10 non-cognates in the dependent spelling measure are presented in Table 2 along with zero-order correlations for word-and person-level features in Table 3. Total accuracy on the dependent English spelling measure showed weak to moderate correlations with Spanish measures of untimed word reading accuracy (.41), untimed decoding accuracy (.29), word spelling (.26), word reading fluency (.23) and decoding fluency (.35). Overall, the average total score on the dependent spelling measure was 41.68 out of 60 words (*SD* = 8.15). The two most difficult words were *recidivism* and *camouflage* (spelled correctly by only 12.9% and 14.5% of the sample, respectively). The two easiest words were *error* and *ritual*, the only words on the task that were spelled correctly by all participants. Across languages, untimed measures of English word reading accuracy, decoding accuracy and decoding fluency were all moderately correlated with measures of Spanish word reading accuracy and decoding fluency. In addition to these two Spanish measures, English word spelling was also correlated moderately with Spanish word spelling. Self-rated spelling abilities in Spanish and English were moderately correlated with each other (.30).

**Table 3.** Word-level feature correlations (*N* = 60) and person-level feature correlations (*N* = 62)

Word-level variables	1	2	3	4	5	6	7	8	9	10	11	12	13
N of Schwas	–												
En frequency	.12	–											
Sp frequency	.19	.61	–										
En N of morphemes	.19*	.06	.17	–									
En length	.35	−.12	.07	.56**	–								
Sp length	.22*	−.13*	.04	.47**	.79**	–							
En OLD20	.27	−.37	−.22	.14	.64**	.51*	–						
En ONS	−.09	.15*	.01	−.21	−.50**	−.36**	−.42**	–					
En PNS	−.10	.35*	.07*	−.22	−.50**	−.42**	−.44**	.54*	–				
En PLD20	.24	−.41	−.26	.23	.64	.56**	.83	−.39*	−.51	–			
Sp ONS	.16	.15	.17	−.07**	−.16**	−.45**	−.21**	.39*	.15**	−.17**	–		
Sp PNS	.20	−.11	.03	.44	.77	.98	.46	−.34	−.43	.54	−.43	–	
OS	−.06*	.13	−.16	.02*	−.18*	−.12**	−.17*	.06	.12*	.08	−.23	−.11*	–
PS	−.33	.14	−.07	−.34	−.41	−.42	−.29	.05	.31	−.24	−.16	−.40	.65

Person-level variables	1	2	3	4	5	6	7	8	9	10	11	12	13	14
Self-rated en spelling skill	–													
Self-rated sp spelling skill	.30*	–												
Sp word reading acc	.09	.30*	–											
Sp decoding acc	.02	.16	.69**	–										
Sp spelling	.04	.31*	.28*	.24	–									
Sp word reading fluency	.12	.19	.56**	.52**	.35*	–								
Sp decoding fluency	.14	.18	.53**	.43**	.34*	.79**	–							
Sp vocab	−.04	.20	.41**	.28*	.43**	.29*	.05	–						
Dependent spelling	.39**	.08	.41**	.29*	.26*	.23	.35*	.19	–					
En word reading acc	.28*	.02	.43**	.31*	.12	.10	.29*	.11	.72**	–				
En decoding acc	.31*	.31*	.62**	.47**	.36*	.33*	.45**	.15	.58**	.51**	–			
En spelling	.37*	.07	.27*	.20	.32*	.18	.36*	.04	.77**	.69**	.47**	–		
En word reading fluency	.07	−.11	−.07	<.01	−.14	.23	.25*	−.14	.20	.14	.17	.20	–	
En decoding fluency	.18	−.01	.27*	.24	.07	.37*	.53**	−.09	.52**	.39**	.53**	.44**	.63**	
En vocab	.02	−.19	−.05	−.06	.05	−.10	−.15	.24	.46**	.48**	.04	.33**	.25	.26*

*Note: M* and *SD* are used to represent mean and standard deviation, respectively. *indicates *p* < .05. **indicates *p* < .001. OLD20 = Orthographic Levenshtein Distance 20; ONS = Orthographic Neighborhood Size; PNS = Phonological Neighborhood Size; PLD20 = Phonological Levenshtein Distance 20; OS = Orthographic Similarity; PS = Phonemic Similarity; En = English; Sp = Spanish; Acc = Accuracy; Vocab = Picture vocabulary. Person-level correlations are only reported for measures that had at least one statistically significant (p < .05) correlation between Spanish and English performance or with the dependent spelling measure.

### 5.3. Unconditional model

A series of crossed random-effects models were run to decompose and model item-level spelling variance associated with persons (*N* = 62) and words (*N* = 60) (see Tables 4 and 5). Probabilities were calculated based on logit estimates from each respective model. In the Unconditional Model (containing only the intercept and random effects), the intercept indicated that the average probability of a correct response across words and participants on the target spelling task was .80. Variance estimates at the person (1.03) and word (3.22) levels suggested that there was significant variance to be explained at both levels in subsequent models.

**Table 4.** Fixed effects predicting probability of correct word spelling responses on complete versus partial dependent spelling task

Fixed effects	Interaction Model (All items included)				Main Effects Model I (Identical cognates removed)			
	Est.	*SE*	*z*	*p*	Est.	*SE*	*z*	*p*
Intercept	**2.10**	**.40**	**5.24**	**<.001**	**1.81**	**.44**	**4.09**	**<.001**
Interactions								
OS × PS	3.77	3.46	1.09	.28	–	–	–	–
**Person factors** ^a^								
En decoding fluency	**.06**	**.02**	**2.99**	**<.01**	**.06**	**.02**	**2.99**	**<.01**
Sp decoding fluency	.03	.02	1.45	.15	.02	.02	1.06	.29
En familiarity	**.05**	**.02**	**2.37**	**.02**	**.06**	**.02**	**2.47**	**.01**
Sp familiarity	<.01	.02	−.14	.89	−.01	.02	−.84	.40
En word reading fluency	**−.02**	**.01**	**−1.98**	**.048**	−.02	.01	−1.59	.11
Sp word reading fluency	−.01	.01	−.49	.63	<.01	.01	−.04	.97
En vocabulary	.04	.03	1.39	.17	.04	.03	1.21	.22
Sp vocabulary	.02	.02	.92	.36	.01	.02	−.74	.46
Sp spelling	.01	.01	.90	.37	.01	.01	1.03	.30
**Word factors** ^b^								
N Schwas	**−.85**	**.30**	**−2.85**	**<.01**	**−.81**	**.31**	**−2.63**	**<.01**
En frequency	**.44**	**.15**	**2.97**	**<.01**	**.47**	**.16**	**2.86**	**<.01**
Sp frequency	**1.31**	**.40**	**2.97**	**<.01**	**1.33**	**.46**	**2.90**	**<.01**
OS	**3.37**	**.94**	**3.57**	**<.001**	1.26	1.26	1.00	.32
PS	−2.59	1.60	−1.62	.11	−1.66	1.94	−.86	.39
**Intercepts**	Variance		Variance explained		Variance		Variance explained	
Person	.41		60.24%		.41		59.84%	
Word	1.44		55.16%		1.20		62.60%	

*Note:* Each of the predictors and respective estimates represent the results from predicting probability of word spelling accuracy from all variables simultaneously (i.e., in the presence of all other word- and person-level predictors in the model). Est. = parameter estimate; SE = standard error; En = English; Sp = Spanish; OS = Orthographic Similarity; PS = Phonemic Similarity.

a Person factors represent aggregate performance by the individual on the measures.

b Word factors represent fixed characteristics of each specific word on the dependent spelling measure.

**Table 5.** Fixed effects predicting probability of correct word spelling responses on dependent spelling task

Fixed effects	Main effects model II				Exploratory interaction model			
	Est.	*SE*	*z*	*p*	Est.	*SE*	*z*	*p*
Intercept	**2.22**	**.39**	**5.71**	**<.001**	**1.79**	**.43**	**4.15**	**<.001**
**Interactions**								
Sp decoding fluency × identical vs non-identical cognate	–	–	–	–	<.01	.01	.52	.60
Sp decoding fluency × non-cognate vs non-identical cognate	–	–	–	–	**.03**	**.01**	**2.21**	**.03**
**Person factors** ^a^								
En decoding fluency	**.06**	**.02**	**2.99**	**<.01**	**.06**	**.02**	**2.97**	**<.01**
Sp decoding fluency	.03	.02	1.45	.15	.02	.02	1.06	.29
En familiarity	**.05**	**.02**	**2.37**	**.02**	**.05**	**.02**	**2.37**	**.02**
Sp familiarity	<.01	.02	−.14	.89	<.01	.02	−.13	.90
En word reading fluency	−.02	.01	−1.98	.05	−.02	.01	−1.96	.05
Sp word reading fluency	−.01	.01	−.49	.63	−.01	.01	−.49	.63
En vocabulary	.04	.03	1.39	.17	.04	.03	1.39	.17
Sp vocabulary	.02	.02	.92	.36	.02	.02	.92	.36
Sp spelling	.01	.01	.90	.37	.01	.01	.89	.37
**Word factors** ^b^								
N of Schwas	**−.83**	**.30**	**−2.77**	**<.01**	**−.81**	**.30**	**−2.72**	**<.01**
En frequency	**.44**	**.15**	**3.01**	**<.01**	**.45**	**.14**	**3.20**	**<.01**
Sp frequency	**1.32**	**.40**	**3.28**	**<.01**	**1.07**	**.38**	**2.82**	**<.01**
OS	**2.94**	**.86**	**3.41**	**<.001**	–	–	–	–
PS	−2.65	1.62	−1.64	.10	−1.91	1.51	−1.26	.21
Identical vs non- identical cognate	–	–	–	–	**1.63**	**.39**	**4.23**	**<.001**
Non-identical vs non-cognate	–	–	–	–	−.78	.61	−1.27	.20
**Intercepts**	Variance		Variance explained		Variance		Variance explained	
Person	.41		60.25%		–		–	
Word	1.47		54.35%		–		–	

*Note:* Each of the predictors and respective estimates represent the results from predicting probability of word spelling accuracy from all variables simultaneously (i.e., in the presence of all other word- and person-level predictors in the model). Est. = parameter estimate; SE = standard error; En = English; Sp = Spanish; OS = Orthographic Similarity; PS = Phonemic Similarity.

a Person factors represent aggregate performance by the individual on the measures.

b Word factors represent fixed characteristics of each specific word on the dependent spelling measure.

### 5.4. Word-level influences

To answer the first research question of whether the interaction of continuous OS and PS measures impact the likelihood of bilinguals’ English word spelling accuracy, the Interaction Model tested the effect of an interaction of OS by PS on item-level spelling accuracy, including person-level variables (i.e., Spanish spelling, English and Spanish measures of decoding fluency, word reading fluency, vocabulary and familiarity with the target spelling items’ spoken pronunciations) and word-level variables (i.e., number of schwas, frequency in both languages, OS and PS) (see Table 4). Higher OS and word frequency (in both Spanish and English) positively predicted spelling accuracy, while words with more schwas showed significantly lower accuracy. However, PS was not a significant predictor of spelling accuracy. Contrary to our first hypothesis, the interaction of OS by PS did not significantly predict item-level spelling accuracy either, demonstrating that the effect of OS on spelling accuracy did not depend on the degree of PS and vice versa. This finding did not change when a variation of this model was run with an interaction of cognateness (instead of OS) by PS or when general English spelling was included in the model (see Supplemental Table S1).

To answer the second research question of whether the facilitatory effects of OS on bilinguals’ English word spelling accuracy is restricted to identical cognates, identical cognates were removed from the outcome variable in the analysis to determine whether the effect of OS would diminish. Given the inclusion of fewer items in total (40 instead of 60), the OS x PS interaction was also removed, resulting in Main Effects Model I (see Table 4). The same person- and word-level variables from the Interaction Model were kept in the model to predict likelihood of non-identical cognate and non-cognate spelling accuracy. As predicted, the effect of OS diminished in this model and was no longer significant, and the PS effect was still non-significant, confirming that OS effects on spelling accuracy are restricted to orthographically identical cognates. No other changes in word-level effects were observed.

### 5.5. Person-level influences

To answer the third research question of which specific literacy-related skills in Spanish and English predict English word spelling accuracy, the same person- and word-level predictors from previous models were entered as fixed effects into Main Effects Model II to simultaneously predict item-level spelling performance of all 60 items, including identical cognates, non-identical cognates and non-cognates (see Table 5). At the person level, significant contributions of general English decoding fluency and familiarity remained unchanged from the previous models, and 60.25% of the person variance was explained. Contrary to our third hypothesis, none of the general Spanish skills emerged as significant predictors of spelling accuracy. While none of the general Spanish literacy measures significantly predicted unique variance in item-level spelling accuracy above and beyond the English measures in any of the models, Spanish measures of decoding fluency and word spelling were individually predictive of item-level spelling accuracy when modeled without any other competing predictors (see Supplemental Table S2). When Spanish predictors were added to a model with only English predictors (i.e., the Main Effects Model II in Table 5), an additional 7% of the person-level variance was explained compared to when only English predictors were in the model (see Supplemental Table S3), demonstrating a small but unique contribution of Spanish literacy skills to the overall person-level variance in item-level spelling accuracy.

### 5.6. Exploratory person-by-word influences

**Figure 2.**
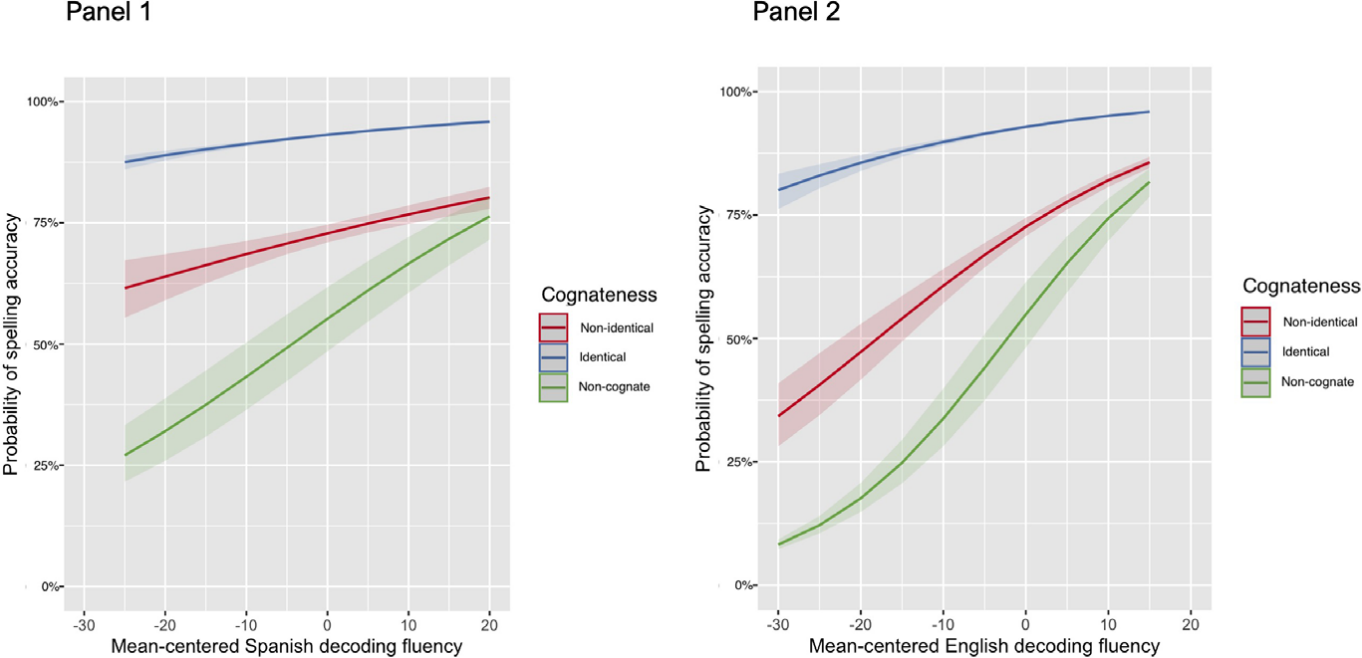
Interactions of cognateness and Spanish versus English decoding fluency in spelling accuracy. *Note:* The x-axis represents mean centered raw scores on the pseudoword reading fluency measures (i.e., decoding) in Spanish (panel 1) and English (panel 2). The y-axis represents probability of correctly spelling a target word in the dependent spelling measure. The legend represents cognateness of a spelling word based on being a non-identical cognate (red line), an identical cognate (blue line), or a non-cognate (green line).

Given the non-significant OS effects on non-identical cognate spelling accuracy (supporting the second hypothesis) and non-significant effects of general Spanish skills on spelling when competing with other English predictors (rejecting the third hypothesis), we examined whether any effect of Spanish decoding fluency may emerge in specific conditions of OS by testing an interaction of cognateness by Spanish decoding fluency (see Table 5). Results showed the same significant person-level and similar word-level contributions (with cognateness replacing OS as a word-level predictor) to item-level spelling accuracy as the previous Interaction Model, plus a significant effect of the contrast between identical and non-identical cognates. This result confirms again that identical cognates were significantly more likely to be spelled correctly compared to non-identical cognates, as we already found in the Main Effects Model I. PS and the contrast between non-identical cognates and non-cognates were the only word-level predictors that did not significantly contribute to item-level spelling accuracy. The interaction of the non-identical cognate versus non-cognate contrast with Spanish decoding fluency was a significant predictor (see first panel of Figure 2), indicating that non-cognates had a lower likelihood of being spelled correctly (~25%–75%) compared to non-identical cognates and the likelihood of non-cognate spelling accuracy increased with higher Spanish decoding fluency. In contrast, participants across the range of Spanish decoding skill had a ~60%–80% probability of correctly spelling non-identical cognates and an ~85%–95% probability of correctly spelling identical cognates, suggesting that higher OS facilitates spelling accuracy regardless of general Spanish decoding skill.

To determine whether this finding was specific to Spanish decoding fluency, we reran the exploratory model with an interaction of cognateness by English decoding fluency (see Supplemental Table S4). Similar results emerged (see second panel of Figure 2), suggesting that the interaction effect of decoding fluency and cognateness is not specific to Spanish or English skill. Simple slope analyses were attempted to probe the slopes of decoding fluency in English versus Spanish at each level of cognateness, but the models could not converge for either exploratory interaction. The convergence issue likely reflects the small number of items in the non-cognate category (*n* = 10) compared to identical cognate (*n =* 20) and non-identical cognate (*n =* 30) categories. However, the exploratory results reported above based on visual inspection of the interaction plots clearly demonstrate how the magnitude of the decoding fluency effect, both in Spanish and English, systematically varies by cognateness, with the weakest effect of fluency on identical cognate spelling and the strongest effect of fluency on non-cognate spelling accuracy.

## 6. Discussion

This study’s purpose was to answer questions about the influence of varying levels of OS and PS and individual differences in language proficiency on spelling accuracy that have not been addressed by previous work with bilingual adults’ spelling. The following discussion situates the current study’s results in the previous literature regarding cognate effects in bilingual populations, beginning with the model results that directly addressed each research question and hypothesis, followed by exploratory model findings.

### 6.1. Word-level influences on spelling accuracy

The non-significant interaction between OS and PS in the Interaction Model did not support the first hypothesis: PS had no distinct effect on spelling accuracy of cognates with low OS versus higher OS. This result is not consistent with Iniesta et al.’s (2021) findings of PS effects on spelling accuracy or previous evidence of low PS inhibiting lexical decision performance in bilingual adults, nor does it support prior findings of “catastrophic” effects of dissimilar phonology in non-identical cognate recognition (Comesaña et al., 2012; Dijkstra et al., 2010; Valente et al., 2018). As a result, while PS has a consistent impact on other processes, like rapid recognition of cognates, it does not seem to consistently impact recall and production of orthographic representations of cognates when accounting for OS. Instead, when number of schwas, OS and frequency are accounted for, the PS effect on spelling accuracy may only be additionally beneficial for similarly spelled words that are more distinct in pronunciations, allowing one to draw on the words’ Spanish pronunciations to help with specific parts of the spelling that are more opaque or inconsistent from pronunciation to spelling in English (i.e., the strategy that was originally hypothesized to be evident in bilinguals’ attempts to recall the irregular spellings of cognates in English).

Given the small partial correlation between PS and number of participants who spelled each cognate correctly when controlling for OS (.11, *p <* .001), it is also likely that these two variables are competing for the same variance when modeled together as word-level predictors of item-level spelling accuracy. One way to address this in future research is to consider the current study’s stimuli selection procedure, which included the calculation of PS using DISC phonetic transcriptions taken from Celex, based on British English pronunciations, rather than American English dialects spoken by the sample. It is possible that if another transcription format that uses single characters for phonemes in both American English and Spanish were to be used, PS values between English and Spanish transcriptions could be recalculated and checked for differences with the values reported in the Appendix. Alternatively, future investigations could follow Schwartz et al.’s (2007) approach to obtaining subjective PS ratings from monolingual English speakers. Additionally, it is worth considering whether measuring onset of spelling attempt (e.g., initial key stroke) and time taken to finish typing the word’s spelling may be more sensitive to capturing PS effects on initial access to or availability of the orthographic representation and sublexical processing of that representation, respectively. Iniesta et al.’s (2021) finding of facilitatory PS effects on both lexical access and sublexical processing in bilingual adults’ word spelling suggests that this approach is more sensitive to PS effects on spelling accuracy of cognates, but it is unclear whether this will hold for cognates that are intentionally selected for having irregular spellings in English.

In contrast to the lack of PS effects, the facilitatory OS effect was clearly consistent across models that included all items from the dependent spelling measure. This finding, however, challenges Iniesta et al.’s (2021) report of no significant OS effects in English spelling accuracy among Spanish-English bilingual adults. This inconsistency may stem from differences in item selection procedures^5^ that led to different proportions of cognates with low OS in the final stimuli composition. While the cognates’ OS range in their study (.2–1) was similar to that of the current study’s stimuli (.38–1), Iniesta and colleagues recognized that a cognate facilitation effect in spelling accuracy may have been masked because one-third of their 104 cognate stimuli had low OS (defined as OS < .70 between Spanish and English). In contrast, the current study’s dependent spelling measure was composed of fewer words overall, with low OS cognates (defined as OS < .50) only comprising 10 of the 50 cognate words. Such differences in how OS was operationalized and the resulting list composition of orthographically identical and non-identical cognates may account for the inconsistent effects of OS reported in accurate spelling of cognates. Given previous findings of stimuli list composition effects on cognate word recognition (Arana et al., 2022; Comesaña et al., 2015), it was relevant to investigate whether the effect of OS would diminish when identical cognates were removed from the model.

The second hypothesis was supported by results of Main Effects Model I: the facilitatory OS effect on spelling accuracy was non-significant when identical cognates were removed from the model. Therefore, the facilitatory OS effect appears to be restricted to spelling of orthographically identical cognates, supporting the notion that representations of identical cognates are processed differently from non-identical cognates (Arana et al., 2022). This finding aligns with the first assumption of the lexical quality hypothesis (LQH): if identical cognates are the words that have the highest likelihood of being encountered in written text and being necessary to recall in one’s own writing experiences (in both languages), then an individual has more exposures to and opportunities to refine these orthographic representations compared to non-identical cognates and non-cognates. This is also supported by the consistently significant and positive contributions of word frequency in both English and Spanish to likelihood of spelling accuracy across all models. In the cases of poor orthographic representations, if there exists a benefit of analyzing an existing phonological representation in Spanish with sublexical units that can match directly onto the more ambiguous sublexical units in the English spelling, then that benefit is restricted to identical cognates. While the redundancy principle may extend to knowledge of how a cognate is pronounced in more than one language, it may only contribute to the formation of a high-quality orthographic representation for identical cognates but not for non-identical cognates. This suggests that for English spelling, any facilitatory effect of redundant phonological representations is likely restricted to bilinguals with proficiency in orthographies that are similar to Spanish in their highly consistent phonology-to-orthography relationships, such as Italian or Finnish. Moreover, if one’s likelihood of correctly spelling non-cognates does not significantly differ from that of non-identical cognates, even those that only differ by a single letter, then the links between phonology and semantics (i.e., those that form during oral vocabulary development and exposure to words beyond reading and writing experience) cannot generally compensate for poor orthographic representations of non-identical cognates and non-cognates when they must be recalled in precise detail from memory.

### 6.2. Person-level influences on spelling accuracy

When Spanish decoding fluency was modeled separately and without other competing predictors, it significantly predicted item-level spelling accuracy in English, suggesting that adults who have stronger Spanish decoding skills (and therefore, more easily activated sublexical representations in Spanish) are more likely to spell English cognates and non-cognates correctly. When competing with general English skills in the models, general Spanish skills explained 6% more of the person-level variance than a model with only English literacy skills, but none of the Spanish predictors significantly contributed to the prediction of item-level spelling accuracy, rejecting the third hypothesis. These results are consistent with previously reported effects of L1 linguistic skills on English spelling diminishing after accounting for English language skills in learners of English as a foreign language, including adults (Russak, 2020) and adolescents (Arfé & Danzak, 2020). The current study’s results also reflect a similar trend in emergent bilingual children from a Spanish-speaking background: general English skills, such as vocabulary (Goodrich et al., 2023; San Francisco et al., 2006) and phonological awareness (Dickinson et al., 2004; Kremin et al., 2019; Sun et al., 2022), are stronger predictors of English reading performance than general abilities in Spanish. We speculate that this would also be replicated with speakers of other heritage languages that are more transparent compared to English as well, but this remains to be tested.

Turning to within-language contributions to spelling accuracy, higher English decoding fluency predicted a higher likelihood of item-level spelling accuracy. This result corroborates previous findings with bilingual adults (Rigobon et al., 2023) and monolingual adults (Mesquita et al., 2022; Ocal & Ehri, 2017a), showing that spelling accuracy is predicted by general decoding abilities in the same language. This pattern also aligns with theories of lexical development (Ehri, 2015; Nation, 2017), which propose that accurate spelling requires deep understanding of language-specific mappings (both phonology-to-orthography and orthography-to-phonology) to develop precise orthographic representations. The contributions of general familiarity with the target words’ pronunciations can best be explained by the notion of continuously “tuning” lexical representations and how it contributes to a skilled reader’s ability to expand their autonomous lexicon over time (see Andrews, 2012; Castles et al., 2007; Hersch & Andrews, 2012). As skilled word readers continue to update and tune representations of individual words with more reading and writing experience, so too evolves their knowledge of orthographic patterns and regularities, along with their understanding of the nuances of a word’s meaning in different contexts. This tuning leads to the addition of more high-quality representations to the lexicon and a more coherent lexical knowledge structure, which is reflected in more accurate word spelling of a wider breadth of words, similarly to how this tuning expands the breadth of words that can be automatically recognized in text (Perfetti & Hart, 2002; Rigobon et al., 2024).

### 6.3. Person-by-word effects on spelling accuracy

Beyond main effects of general English literacy skills on spelling accuracy, the exploratory interaction model’s results partially support the third hypothesis. The significant interaction of Spanish decoding fluency and cognateness shows that the ability to apply sublexical knowledge from Spanish appears to influence English word spelling differently for cognates versus non-cognates. More specifically, the likelihood of non-cognate and non-identical cognate spelling accuracy increases with higher Spanish decoding fluency, while likelihood of identical cognate spelling accuracy does not vary greatly by Spanish decoding skill. This result supports the notion that availability of phonological representations which are recoverable from more transparent mappings of phonology-to-orthography in not just one, but two languages, can aid accurate spelling, but perhaps general knowledge of these mappings provides more support in non-cognate and non-identical cognate spelling compared to identical cognate spelling. Following the assumption that bilinguals have generally fewer exposures to non-cognates compared to cognates (e.g., Gollan et al., 2008 weaker links hypothesis), non-cognates should have a higher likelihood of being represented with lower quality in the bilingual lexicon, likely leading individuals to rely on a strategy to supplement a higher number of partial or imprecise orthographic representations of non-cognates compared to cognates. Based on participants’ reflections on general strategies they used to complete the dependent spelling task (see Table 1), 45% of the sample reported visualizing the word’s spelling in Spanish and 38% reported attempting to silently sound out the target word letter by letter in Spanish,^6^ suggesting that further exploration (beyond the current paper’s scope) of cross-linguistic strategy use and total performance on the spelling task may potentially shed light on bilingual adults’ ability to successfully apply strategies using general knowledge of sublexical units in Spanish to recall or generate word spellings in English for non-cognates and non-identical cognates.

Additionally, as highlighted in Figure 2, the difference in likelihood of spelling accuracy between non-identical cognates and non-cognates was largest for participants with weaker decoding skills, meaning that poorer Spanish decoders had a higher probability of correctly spelling non-identical cognates with otherwise similar features (e.g., length, frequency in English and Spanish) compared to the non-cognates. This suggests that for bilinguals with poorer sublexical representations in Spanish, the cognate facilitation effect in spelling is not purely driven by identical cognates, whereas for bilinguals with stronger Spanish decoding skills, the cognate facilitation effect in spelling is indeed restricted to identical cognates. This finding supports the LQH’s assumption that high-quality word representations established with redundant phonological representations can boost the probability of accurate spelling over time (Perfetti & Hart, 2001, 2002), but this redundancy with pronunciations derived from Spanish is likely more important for poorer Spanish decoders when attempting to spell non-identical cognates and non-cognates.

A separate analysis showed that an interaction of English decoding fluency and cognateness was also significant, which suggests that the general (rather than language-specific) ability to apply knowledge of sublexical relationships is most impactful for spelling of non-cognates, regardless of which language that knowledge is drawn from. The weak effect of decoding fluency (in both languages) on identical cognate spelling highlights the special roles of identical spelling and shared meaning in facilitating spelling accuracy such that (a) individuals do not need to rely on general knowledge of sublexical units to spell these cognates correctly as these words are already represented with high quality in the bilingual lexicon or (b) strategies applied to supplement poor representations of these words tend to be more effective. This facilitation seems to be experienced by the whole sample, regardless of their Spanish literacy skills, expanding evidence of the “special status” of identical cognates in lexical decision (Comesaña et al., 2015; Dijkstra et al., 2010; Guasch et al., 2017; Peeters et al., 2013) to the task of word spelling. Once again, we speculate that this finding would hold true for bilingual adults who are heritage speakers of other languages, such as Italian, which is similarly transparent to Spanish and shares cognates with English that range in their orthographic and phonological similarity between translation equivalents.

However, it is worth considering how these effects may differ for other profiles of bilingual adults, such as late learners of English who are monolingual in a more transparent orthography like Spanish for a longer period of early literacy development (e.g., only Spanish classroom instruction for K-5). We could speculate that for this language background, bilingual adults might be more sensitive to cognate effects in their spelling of non-identical cognates if their acquisition of English orthography and phonology has relied on more systematic comparisons of English and Spanish language systems throughout formal language and literacy instruction. This is just one of many possible avenues for future investigations of cognate effects on the spelling performance of different subgroups of bilingual adults.

Additionally, we must acknowledge that among the study’s limitations is the difference in structure of the fluency tasks between English and Spanish: both the sight word reading (i.e., real word reading fluency) and pseudoword reading efficiency (i.e., decoding fluency) subtests from the TOWRE-2 increase in difficulty as the participant reads further down the lists, whereas items are not ordered by increasing difficulty in the Spanish fluency tests. On average, the English word reading fluency subtest also contains longer items (8.6 letters) than the Spanish subtest from the LEE (6.19 letters long). Spanish fluency tests were administered to allow participants to attempt all items as quickly as possible, attempts were timed, and fluency scores were calculated as number of words read correctly per 45 seconds (an effort to create equivalent Spanish fluency scores to raw scores on the 45-second limited English fluency tests), but it is possible that the difference in assessment design could have influenced participants’ performance. Given our sample’s lack of formal instruction in Spanish literacy, we believe any timed test of Spanish word reading or decoding would have likely been more challenging than in English (which is evident in the wider ranges and lower average scores of performance on the Spanish measures compared to the English measures). Still, future investigations may benefit from administering measures in each language that are more closely matched in their order of item difficulty and other item features, especially for capturing information about participant samples with more extensive literacy experience in both languages.

## 6.4. Conclusion

This study examined how cross-linguistic overlap of words and literacy skills in Spanish and English can contribute to spelling accuracy, particularly for irregular spellings in a more opaque orthography. We found significant influences of word features, including OS and frequency of appearing in both English and Spanish texts on spelling accuracy. Significant person-level influences included an individual’s general English decoding fluency and general familiarity with the target spelling words, while general Spanish decoding and spelling skills influenced spelling accuracy to a small extent. Contrary to our prediction, we did not find any evidence of OS effects on spelling accuracy varying by PS. However, evidence of the “special status” of orthographically identical cognates previously reported in faster and more accurate lexical decision responses (Comesaña et al., 2015; Dijkstra et al., 2010; Guasch et al., 2017; Peeters et al., 2013) was extended here to the task of word spelling, suggesting that identical cognates tend to be stored with higher quality than non-identical cognates and non-cognates. As such, this is the first study to show that the facilitatory OS effect on cognate spelling is restricted to identical cognates. The significant two-way interactions of cognateness and decoding fluency (in both Spanish and English) also highlighted that the strength or availability of sublexical representations in either language is most impactful on bilingual adults’ likelihood of accurate non-cognate spelling, suggesting the possibility of bilingual individuals successfully applying strategies based on knowledge of both Spanish and English to supplement recall of any lower quality orthographic representation in English word spelling, not just cognates.

## Supporting information

10.1017/S1366728926101126.sm001 Rigobon et al. supplementary material Rigobon et al. supplementary material

## Data Availability

Data are available upon email request to valeria.rigobon@acu.edu.au.

## Footnotes

1 In the cited studies, PS refers to **phonological** similarity, while PS in the current study more specifically refers to **phonemic** similarity.

2 If a participant repeated a different word than the target word, they were directed to replay the audio file once and repeat the word aloud. If they still did not repeat the correct word, the tester said the correct word and the participants had one more opportunity to repeat the word before proceeding with the task.

3 Following Schepens et al.’s (2013) recommendations for cross-language comparisons of cognate features, DISC was used for item transcription because it is an IPA-based coding scheme that represents IPA symbols as single characters, allowing for the transcriptions to be analyzed without recoding any symbols or making multiple substitutions for special characters (which is typical when computing PS between traditional IPA transcriptions).

4 For example, in the interaction model fixed effects reported in Table 4, grand mean-centering the continuous predictors allows one to interpret the intercept of 1.96 as the expected log-odds of a correct response for a participant with an average score on the English word familiarity measure responding to a word with average frequency. The coefficient of .06 for English familiarity (a person-level predictor) then represents how much the log-odds increase for each unit above the average familiarity level (holding word frequency constant), and the coefficient of .37 for word frequency represents how much the log-odds increase for each unit above average frequency (holding person familiarity constant), making both effects directly interpretable around meaningful reference points.

5 Cognate stimuli in Iniesta et al.’s (2021) study were extracted from Schwartz et al.’s (2007) study.

6 In the final survey, participants could choose to report more than one strategy that they used during the spelling task, so there is some overlap between those who reported each of the Spanish-based strategies mentioned here.

## Supplementary material

The supplementary material for this article can be found at http://doi.org/10.1017/S1366728926101126.

## Appendix

### A. Dependent spelling measure items

**Table:**

	Dependent Spelling Words (*N* = 60)				Cross-Linguistic Similarity^a^		% of Sample Correctly Spelled
	*English*	*DISC* ^b^ *Phonetic Transcription*	*Spanish*	*DISC Phonetic Transcription*	*Orthographic*	*Phonemic*	
O=P+ Cognates (Identical)	error	Er@R	error	ErOR	1.00	0.75	100%
	extension	IkstEnSH	extensión	EkstEnsiOn	1.00	0.50	91.94%
	iguana	Igw#n@	iguana	igwAnA	1.00	0.50	93.89%
	marsupial	m#supj@l	marsupial	mARsUpjAl	1.00	0.56	69.35%
	mosquito	m@skit5	mosquito	mOskitO	1.00	0.57	96.77%
	salmon	s{m@n	salmón	sAlmOn	1.00	0.50	93.55%
	sepia	sipj@	sepia	sEpjA	1.00	0.60	58.06%
	suspension	s@spEnSH	suspensión	sUspEnsiOn	1.00	0.50	93.55%
	talisman	t{lIzm@n	talisman	tAlismAn	1.00	0.50	58.06%
	tandem	t{nd@m	tandem	tAndEm	1.00	0.67	54.84%
O=P− Cognates (Identical)	adhesion	@dhiZH	adhesión	AdEsiOn	1.00	0.29	93.55%
	arsenal	#s@nP	arsenal	ARsEnAl	1.00	0.29	79.03%
	cafeteria	k{fIt7r7	cafeteria	kAfEtERiA	1.00	0.33	96.77%
	dispersion	dIsp3SH	dispersión	dispERsiOn	1.00	0.30	80.65%
	expulsion	IkspVlSH	expulsión	EkspUlsiOn	1.00	0.40	83.87%
	ingestion	In_EsJ@n	ingestión	inxEstiOn	1.00	0.44	93.55%
	lateral	l{t@r@l	lateral	lAtERAl	1.00	0.43	90.32%
	orangutan	$r{N@t{n	orangutan	ORAngUtAn	1.00	0.22	51.61%
	ritual	rIJ9l	ritual	ritwAl	1.00	0.33	100%
	transfusion	tr{nsfjuZH	transfusión	tRAnsfUsiOn	1.00	0.36	96.77%
O+P+ Cognates (Non-identical)	antidote	{ntId5t	antidoto	AntidOtO	0.88	0.50	80.65%
	aqueduct	{kwIdVkt	acueducto	AkwEdUktO	0.78	0.56	38.71%
	assault	@s$lt	asalto	AsAltO	0.57	0.50	74.19%
	chameleon	k@milj@n	camaleon	kAmAlEOn	0.78	0.50	43.55%
	consultation	kQns@lt1SH	consulta	kOnsUltA	0.67	0.50	88.71%
	exploitation	Ekspl4t1SH	explotación	EksplOtATiOn	0.83	0.50	85.48%
	gazelle	g@zEl	gacela	gATElA	0.57	0.50	66.13%
	glutton	glVtH	glotón	glOtOn	0.71	0.50	50%
	mollusk	mQl@sk	molusco	mOlUskO	0.57	0.57	25.81%
	polyp	pQlIp	pólipo	pOlipO	0.67	0.50	27.42%
O+P− Cognates (Non-identical)	accelerator	@ksEl@r1t@R	acelerador	ATElERAdOR	0.82	0.27	74.19%
	acupuncture	{kjUpVNkJ@R	acupuntura	AkUpUntURA	0.82	0.27	24.19%
	annihilation	@"n2@l1SH	aniquilación	AnikilATiOn	0.67	0.09	20.97%
	camouflage	k{m@fl#Z	camuflage	kAmUflAxE	0.80	0.44	14.52%
	exhumation	Ekshjum1SH	exhumación	EksUmATiOn	0.90	0.30	25.81%
	genocide	_En5s2d	genocidio	xEnOTidjO	0.78	0.33	88.71%
	hippopotamus	hIp@pQt@m@s	hipopótamo	ipOpOtAmO	0.75	0.36	37.10%
	indulgence	ndVl_@ns	indulgencia	indUlxEnTjA	0.82	0.36	80.65%
	passage	p{sI_	pasaje	pAsAxe	0.71	0.40	98.39%
	pivot	pIv@t	pivote	pibOtE	0.83	0.33	88.71%
	scrutiny	skrutInI	escrutinio	EskRUtinjO	0.70	0.40	70.97%
O−P− Cognates (Non-identical)	amphibian	{mfIb7n	anfibio	AnfibjO	0.44	0.29	85.48%
	appearance	@p7r@ns	apariencia	ApARjEnTjA	0.40	0.20	80.65%
	consolation	kQns@l1SH	consuelo	kOnswElO	0.45	0.33	51.51%
	ephemeral	IfEm@r@l	efímero	EfimERO	0.44	0.25	27.42%
	isolation	2s@l1SH	aislamiento	AjslAmjEntO	0.45	0.09	96.77%
	mammal	m{mP	mamífero	mAmifERO	0.38	0.25	87.10%
	occupancy	QkjUp@nsI	ocupación	OkUpATiOn	0.44	0.22	93.55%
	quantity	kwQnt@tI	cantidad	kAntidAd	0.38	0.25	96.77%
	recidivism	rIsIdIvIz@m	reincidencia	rEjnTidEnTjA	0.42	0.08	12.90%
Non-Cognates	appraisal	\@pr1zP	tasación	tAsATiOn	0.11	0.00	72.58%
	behemoth	bIhim@T	gigante	xigAntE	0.13	0.50	24.19%
	burglary	b3gl@rI	robo	rObO	0.13	0.00	58.06%
	counterfeit	k6nt@fIt	falsificación	fAlsifikATiOn	0.08	0.08	51.61%
	disguised	dIsg2zd	disfrazado	disfrATAdO	0.40	0.30	85.48%
	foreigner	fQr@n@R	extranjero	EkstRAnxERO	0.20	0.18	75.81%
	gruesome	grus@m	horrible	OriblE	0.13	0.17	69.35%
	heirloom	8lum	reliquia	rElikjA	0.13	0.14	64.52%
	nourishment	\nVrISm@nt	alimentación	AlimEntATiOn	0.08	0.00	83.87%
	unscathed	Vnsk1Dd	indemne	indEmnE	0.22	0.14	64.52%

*Note: All participants were presented with an audio recording of the target English spelling words in randomized order for the English familiarity and target spelling tasks. All participants were also presented with an audio recording list of the original Spanish words in randomized order for the Spanish familiarity task.*

a Values for both orthographic and phonological similarity were reverse coded from Normalized Damerau-Levenshtein Distance (NDLD) to represent similarity values with higher values indicating higher similarity rather than distance. NDLD values were calculated based on the minimum number of operations required to transform the Spanish character string into the English character string. Accented letters in Spanish were not treated as different characters in calculating the orthographic NDLD values, and a transposition (i.e., two letters appearing consecutively but in different letter positions between translation equivalents) is calculated as only one operation rather than two (unlike Normalized Levenshtein Distance [NLD] values that consider a transposition to be two operations and result in slightly larger distance values, such as those provided by Guasch et al.’s 2013 NIM software).

b Phonological transcriptions were calculated using DISC transcriptions of the English words from the Celex database (Baayen et al., 1993) and Spanish translation equivalents from the B-PAL database (Davis & Perea, 2005). DISC is an IPA-based coding scheme that represents IPA symbols as single characters, which allows for the transcriptions to be analyzed without needing to recode any symbols or make multiple substitutions for special characters (which is typical when computing phonemic similarity between traditional IPA transcriptions).
