# Supplementary material for: Investigating individual differences in adult bilinguals’ spelling of cognates: An analysis of cross-linguistic effects
Source: Biling (Camb Engl). 2026 Apr 15:1–18. Online ahead of print. doi: 10.1017/S1366728926101126 (PMC13107191; doi:10.1017/S1366728926101126)
Supplement: Rigobon et al. supplementary material [file S1366728926101126sup001.pdf]

Supplemental Files

Link to see downloadable file with analysis code on OSF:
https://osf.io/36q2u/overview?view_only=5bd4fb78ed9c4a5886ca398bedebbce2

Table S1.
Fixed effects (person-level, word-level, and alternative word interaction) predicting probability of correct word spelling responses on dependent spelling task.

	Cognate Status X PS Model				Alternate OS X PS Model			
Fixed effects	Est.	*SE*	*z*	*p*	Est.	*SE*	*z*	*p*
**Intercept**	**1.76**	**.45**	**3.90**	**<.001**	**2.13**	**.40**	**5.37**	**<.001**
**Interactions**								
OS X PS	–	–	–	–	3.77	3.49	1.08	.28
Identical vs Non-Identical Cognate X PS	-.80	2.84	-.28	.78	–	–	–	–
Non-Cognate vs Non-Identical Cognate X PS	.46	4.47	.10	.92	–	–	–	–
**Person factors**[a]								
En Decoding Fluency	**.06**	**.02**	**2.99**	**<.01**	**.04**	**.02**	**2.51**	**.01**
Sp Decoding Fluency	.03	.02	1.45	.15	.01	.02	.40	.69
En Familiarity	**.05**	**.02**	**2.37**	**.02**	.02	.02	1.01	.31
Sp Familiarity	<.01	.02	-.14	.89	-.01	.01	-.53	.60
En Word Reading Fluency	**-.02**	**.01**	**-1.98**	**.048**	**-.02**	**.01**	**-2.15**	**.03**
Sp Word Reading Fluency	-.01	.01	-.49	.63	<.01	.01	.24	.81
En Vocabulary	.04	.03	1.39	.17	.03	.02	1.36	.17
Sp Vocabulary	.02	.02	.92	.36	.02	.01	1.66	.10
En Spelling	–	–	–	–	**.17**	**.03**	**5.96**	**<.001**
Sp Spelling	.01	.01	.90	.37	<-.01	.01	-.41	.68
**Word factors**[b]								
N of Schwas	**-.79**	**.32**	**-2.44**	**.01**	**-.86**	**.30**	**-2.85**	**<.01**

En Frequency	**.46**	**.14**	**3.10**	**<.01**	**.44**	**.15**	**2.97**	**<.01**
Sp Frequency	**1.07**	**.39**	**2.76**	**<.001**	**1.31**	**.40**	**3.29**	**<.01**
OS	–	–	–	–	**3.37**	**.95**	**3.56**	**<.001**
PS	-1.65	1.82	-.91	.36	-2.60	1.61	-1.62	.11
Identical vs Non-Identical Cognate	**1.67**	**.42**	**3.99**	**<.001**	–	–	–	–
Non-Identical vs Non-Cognate	-.61	1.26	-.48	.63	–	–	–	–

**Intercepts**	Variance	Variance Explained	Variance	Variance Explained
Person	.41	60.23%	.21	79.83%
Word	1.31	59.27%	1.44	55.19%

Note. Each of the predictors and respective estimates represent the results from predicting probability of word spelling accuracy from all variables simultaneously (i.e., in the presence of all other word- and person-level predictors in the model).
Est.= parameter estimate; SE = standard error; En = English; Sp = Spanish; OS = Orthographic Similarity; PS = Phonemic Similarity.
[a]Person factors represent aggregate performance by the individual on the measures. [b]Word factors represent fixed characteristics of each specific word on the dependent spelling measure.

Table S2.

Fixed effects (Spanish only) predicting probability of correct word spelling responses on dependent spelling task in separate single predictor models

Fixed effects	Est.	*SE*	*z*	*p*
**Intercept**	**1.39**	**.27**	**5.20**	**<.001**
Decoding Fluency	**.04**	**.01**	**2.80**	**.01**
**Intercept**	**1.38**	**.27**	**5.12**	**<.001**
Familiarity	.03	.02	1.73	.08
**Intercept**	**1.39**	**.27**	**5.15**	**<.001**
Word Reading Fluency	.02	.01	1.77	.08
**Intercept**	**1.39**	**.27**	**5.15**	**<.001**
Vocabulary	.03	.02	1.58	.11
**Intercept**	**1.36**	**.27**	**5.07**	**<.001**
Spelling	**.03**	**.01**	**2.30**	**.02**

**Models**	Variance	Variance Explained
Decoding Fluency	.89	13.66%
Familiarity	.98	4.97%
Word Reading Fluency	.97	5.90%
Vocabulary	.98	4.34%
Spelling	.94	8.61%

Note. Each of the predictors and respective estimates represent the results from predicting probability of word spelling accuracy from all variables simultaneously (i.e., in the presence of all other word- and person-level predictors in the model).

Est.= parameter estimate; SE = standard error; En = English; Sp = Spanish; OS = Orthographic Similarity; PS = Phonemic Similarity.

[a]Person factors represent aggregate performance by the individual on the measures. [b]Word factors represent fixed characteristics of each specific word on the dependent spelling measure.

Table S3.

Fixed effects (English only) predicting probability of correct word spelling responses on dependent spelling task

Fixed effects	Est.	*SE*	*z*	*p*
**Intercept**	**2.24**	**.39**	**5.73**	**<.001**
**Person factors**[a]				
Decoding Fluency	**.08**	**.02**	**4.63**	**<.001**
Familiarity	**.05**	**.02**	**2.35**	**.02**
Word Reading Fluency	**-.02**	**.01**	**-2.58**	**.01**
Vocabulary	.04	.03	1.30	.19
**Word factors**[b]				
N of Schwas	**-.83**	**.30**	**-2.77**	**<.01**
En Frequency	**.44**	**.15**	**3.01**	**<.01**
Sp Frequency	**1.32**	**.40**	**3.28**	**<.01**
OS	**2.94**	**.86**	**3.41**	**<.001**
PS	-2.65	1.61	-1.64	.10

**Intercepts**	Variance	Variance Explained
Person	.48	53.19%
Word	1.47	54.33%

Note. Each of the predictors and respective estimates represent the results from predicting probability of word spelling accuracy from all variables simultaneously (i.e., in the presence of all other word- and person-level predictors in the model).

Est.= parameter estimate; SE = standard error; En = English; Sp = Spanish; OS = Orthographic Similarity; PS = Phonemic Similarity.

[a]Person factors represent aggregate performance by the individual on the measures. [b]Word factors represent fixed characteristics of each specific word on the dependent spelling measure.

The variance estimates and variance explained are presented here for comparison with the person variance estimated and variance explained in Main Effects Model II (Table 5), which includes Spanish predictors.

Table S4.
Fixed effects (person-level, word-level, and alternate exploratory interaction) predicting probability of correct word spelling responses on dependent spelling task

Fixed effects	Est.	*SE*	*z*	*p*
**Intercept**	**1.80**	**.43**	**4.17**	**<.001**
**Interactions**				
En Decoding Fluency X Identical vs Non-Identical Cognate	-.01	.01	-1.09	.27
En Decoding Fluency X Non-Cognate vs Non-Identical Cognate	**.03**	**.02**	**2.10**	**.04**
**Person factors**[a]				
En Decoding Fluency	**.05**	**.02**	**2.72**	**.01**
Sp Decoding Fluency	.03	.02	1.44	.15
En Familiarity	**.05**	**.02**	**2.34**	**.02**
Sp Familiarity	<-.01	.02	-.11	.91
En Word Reading Fluency	**-.02**	**.01**	**-1.97**	**.049**
Sp Word Reading Fluency	-.01	.01	-.48	.63
En Vocabulary	.04	.03	1.39	.17
Sp Vocabulary	.01	.02	.90	.37
Sp Spelling	.01	.01	.90	.37
**Word factors**[b]				
N of Schwas	**-.82**	**.30**	**-2.73**	**<.01**
En Frequency	**.45**	**.14**	**3.20**	**<.01**
Sp Frequency	**1.07**	**.38**	**2.81**	**<.01**
Identical vs Non-Identical Cognate	**1.59**	**.39**	**4.14**	**<.001**
Non-Identical vs Non-Cognate	-.78	.61	-1.28	.20
PS	-1.91	1.51	-1.26	.21

Note. Each of the predictors and respective estimates represent the results from predicting probability of word spelling accuracy from all variables simultaneously (i.e., in the presence of all other word- and person-level predictors in the model).

Est.= parameter estimate; SE = standard error; En = English; Sp = Spanish; OS = Orthographic Similarity; PS = Phonemic Similarity.

[a]Person factors represent aggregate performance by the individual on the measures. [b]Word factors represent fixed characteristics of each specific word on the dependent spelling measure.
